# Supplementary material for: Physicochemical Properties and Anticoagulant Activity of Purified Heteropolysaccharides from Laminaria japonica
Source: Molecules. 2022 May 8;27(9):3027. doi: 10.3390/molecules27093027 (PMC9102426; doi:10.3390/molecules27093027)
Supplement: Supplementary file 1 [file molecules-27-03027-s001.zip › molecules-1684802-supplementary.pdf]

Supporting Information

Physicochemical properties and anticoagulant activity of purified heteropolysaccharides from *Laminaria japonica*

Tingting Li [1,†], Haiqiong Ma [1,†], Hong Li [1], Hao Tang [1], Jinwen Huang [1], Shiying Wei [1], Qingxia Yuan [1], Xiaohuo Shi [2], Chenghai Gao [1], Shunli Mi [1], Longyan Zhao [1,*], Shengping Zhong [1,*] and Yonghong Liu [1,*]

[1] Institute of Marine Drugs, Guangxi University of Chinese Medicine, Nanning 530200, China; li15578909861@126.com (T.L.); MHQ18878839254@163.com (H.M.); hongli12212022@163.com (H.L.); yaoxuetanghao@outlook.com (H.T.); huangjinwen1127@163.com (J.H.); wsy980915@163.com (S.W.); qingxiayuan@163.com (Q.Y.); gaochh@gxtcmu.edu.cn (C.G.); mishunli@126.com (S.M.)

[2] Key Laboratory of Precise Synthesis of Functional Molecules of Zhejiang Province, School of Science, Westlake University, Hangzhou 310024, China; shixiaohuo@westlake.edu.cn (X.S.)

* Correspondence: longyanzhao@gmail.com (L.Z.); shpzhong@foxmail.com (S.Z.); yonghongliu@scsio.ac.cn (Y.L.)

† These authors contributed equally to this work.

Citation: Li, T.; Ma, H.; Li, H.; Tang, H.; Huang, J.; Wei, S.; Yuan, Q.; Shi, X.; Gao, C.; Mi, S.; et al. Physicochemical Properties and Anticoagulant Activity of Purified Heteropolysaccharides from Laminaria japonica. Molecules 2022, 27, 3027 https://doi.org/10.3390/molecules27093027

Academic Editors: Lesław Juszczak and Janusz Kapusniak

Received: 31 March 2022

Accepted: 5 May 2022

Published: 8 May 2022

Publisher's Note: MDPI stays neutral with regard to jurisdictional claims in published maps and institutional affiliations.

Copyright: © 2022 by the authors. Submitted for possible open access publication under the terms and conditions of the Creative Commons Attribution (CC BY) license (https://creativecommons.org/licenses/by/4.0/).

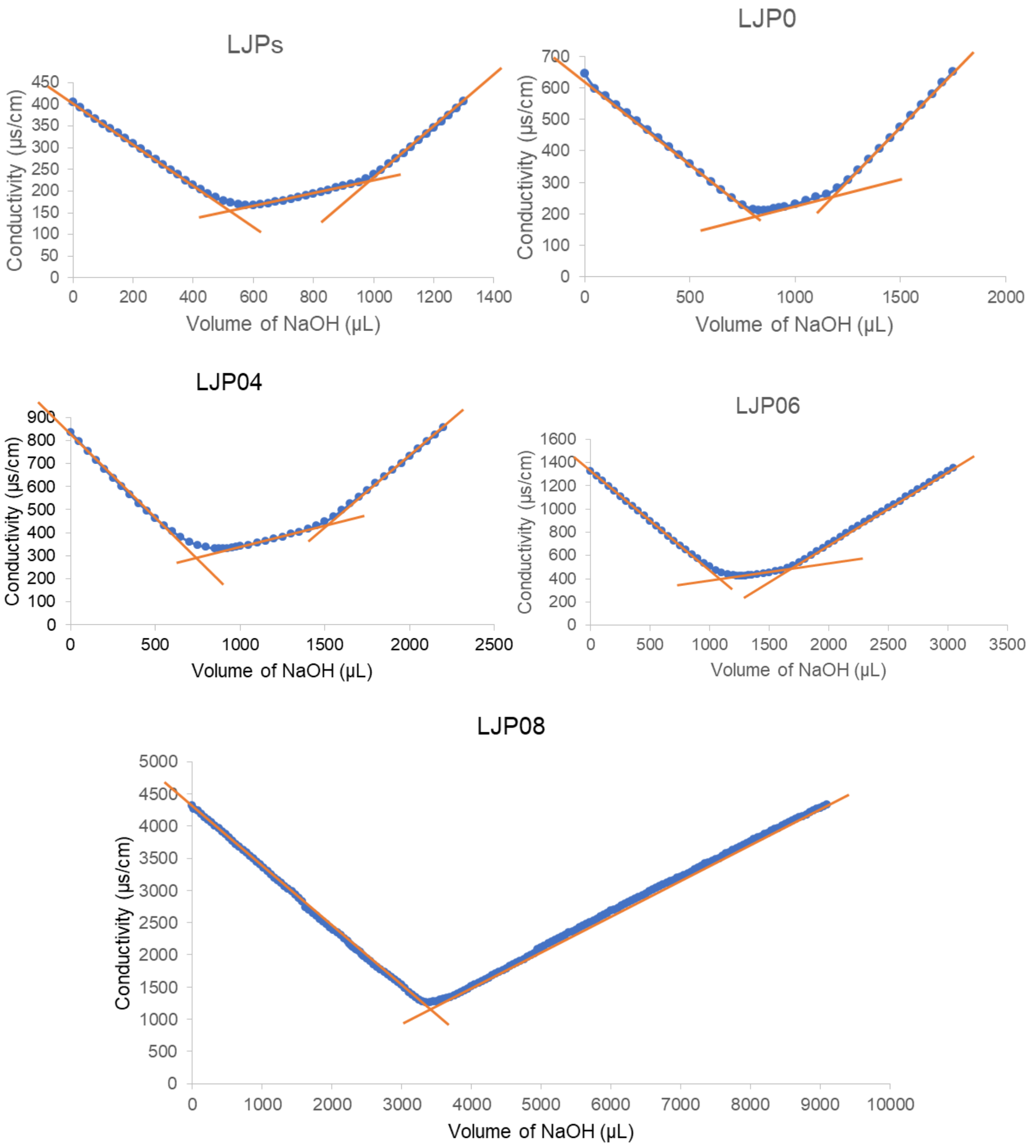

Figure S1. Conductimetric titration curves of LJPs, LJP0, LJP04, LJP06, and LJP08

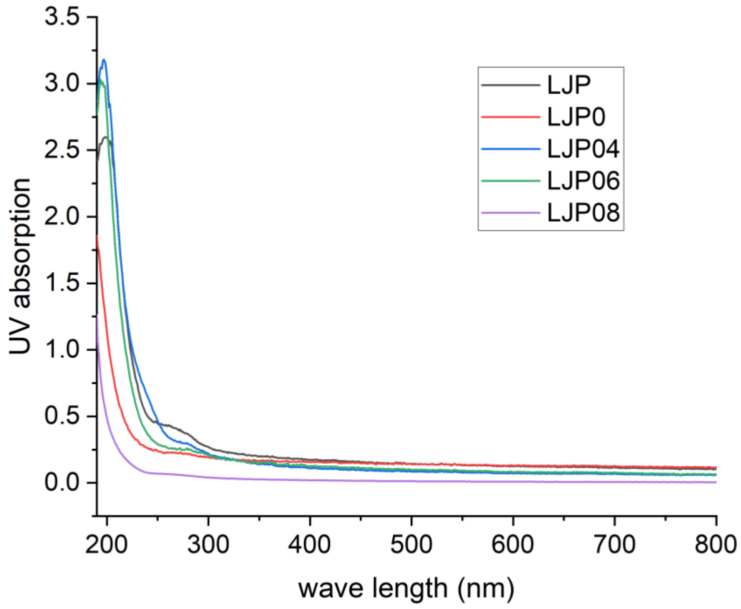

Figure S2. UV spectrum of LJPs and its purified fractions
